# Public Preference and Priorities for Including Vaccines in China’s National Immunization Program: Discrete Choice Experiment

**DOI:** 10.2196/57798

**Published:** 2024-11-14

**Authors:** Lingli Zhang, Xin Li, Jiali Chen, Xiaoye Wang, Yuyang Sun

**Affiliations:** 1 School of International Pharmaceutical Business, China Pharmaceutical University, Nanjing, China; 2 Department of Pharmacy, The Second People's Hospital of Changzhou, The Third Affiliated Hospital of Nanjing Medical University, Changzhou, China; 3 School of Health Policy and Management, Nanjing Medical University, Nanjing, China; 4 School of Pharmacy, Nanjing Medical University, 101 Longmian Avenue, Jiangning District, Nanjing, 211166, China, 86 025 86868467; 5 Center for Global Health, School of Public Health, Nanjing Medical University, Nanjing, China

**Keywords:** discrete choice experiment, national immunization program, vaccines, vaccination, immunization, public preferences, China, mixed logit model, heterogeneity, varicella, public health, infectious diseases

## Abstract

**Background:**

Several important vaccines, such as the *Haemophilus influenzae* type b vaccine, rotavirus vaccine, pneumococcal conjugate vaccine, and influenza vaccine, have not been included in China’s National Immunization Program (NIP) due to a prolonged absence of updates and limited resources. Public engagement could identify concerns that require attention and foster trust to ensure continuous support for immunization.

**Objective:**

This study aimed to identify public preferences for vaccine inclusion in the NIP and to determine the desired vaccine funding priorities in the Chinese population.

**Methods:**

A dual-response discrete choice experiment was utilized to estimate the relative importance of 6 attributes, including incidence of vaccine-preventable diseases, mortality of vaccine-preventable diseases, vaccine effectiveness, vaccine cost, vaccinated group, and vaccine coverage. Participants were recruited through the Wenjuanxing platform using a census-based quota sample of the nationwide population aged 18 years and older. A mixed logit model was used to estimate the coefficient of attribute preferences and predict the selection probability. Subgroups and interaction effects were analyzed to examine the heterogeneity in preferences.

**Results:**

In total, 1258 participants completed the survey, of which 880 were involved in the main analysis and 1166 in the sensitivity analysis. The relative importance and model estimates of 2 attributes, vaccine cost and vaccination group, varied between the unforced- and forced-choice settings. All 6 vaccine attributes significantly influenced the preferences for vaccine inclusion, with vaccine effectiveness and coverage as the most important factors, followed by the vaccinated group and mortality of vaccine-preventable diseases in the unforced-choice settings. The top vaccines recommended for China’s NIP included the varicella vaccine, *Haemophilus influenzae* type b vaccine, enterovirus 71 vaccine, and influenza vaccine for preschoolers and school-aged children. The current analysis also revealed distinct preference patterns among different subgroups, such as gender, age, education, and income. The interaction analysis indicated that the region and health status of participants contribute to preference heterogeneity.

**Conclusions:**

Public preferences for including vaccines in the NIP were primarily influenced by vaccine effectiveness and coverage. The varicella vaccine should be prioritized for inclusion in the NIP. The public preferences could provide valuable insights when incorporating new vaccines in the NIP.

## Introduction

Immunization is crucial in controlling and preventing infectious diseases, averting 3.5‐5 million deaths annually from diseases such as diphtheria, tetanus, pertussis, influenza, and measles [1]. The Immunization Agenda 2030, developed by the World Health Organization (WHO), highlights the necessity of universal accessibility to effective and efficient immunization services worldwide [2]. China’s National Immunization Program (NIP) has achieved remarkable success through a substantial increase in vaccine coverage to over 95% and a significant reduction in the burden of associated diseases [3]. However, no vaccine has been added to the NIP since 2008. The National Immunization Advisory Committee, established in 2017, has not made any recommendations despite being responsible for providing evidence-based recommendations for new vaccine incorporations into the NIP. Additionally, the National Immunization Advisory Committee membership consists solely of internally selected experts, and therefore lacks consumer representation [3].

The *Haemophilus influenzae* type b (Hib) vaccine, rotavirus vaccine, pneumococcal conjugate vaccine (PCV), influenza vaccine, human papillomavirus (HPV) vaccine, and varicella vaccine have all been recommended by the WHO for routine immunization [4]. However, these crucial vaccines have not been included in China’s NIP due to a prolonged absence of updates. Although the NIP vaccines are mandatory and provided free of charge to all eligible recipients, non-NIP vaccines in China are voluntarily administered and self-funded, leading to low coverage and substantial inequalities in terms of immunization in the country [5].

Incorporating vaccines into the NIP is a complex decision-making process that requires consideration of many aspects, including the diseases targeted by the vaccine, vaccine characteristics, and capacity of the health system [6]. Numerous vaccines have been proposed for inclusion in China’s NIP based on their cost-effectiveness or ability to reduce the burden associated with the targeted diseases [7-13]. However, actioning these proposals has been hindered by the government’s limited financial resources. Unlike many countries where vaccines are covered by health insurance, vaccines in China are funded by the government via the NIP [14]. The central government procures and funds the NIP vaccines, while local governments finance the immunization services. Despite increasing government investment in the NIP, the financing remains insufficient, which highlights the necessity of prioritizing various vaccines.

Prior studies have investigated the prioritization of vaccines for inclusion in China’s NIP, relying on expert perspectives [14,15]. Although expert opinions are of utmost importance, vaccine incorporation into the NIP should also consider public needs. This is because the public is a key stakeholder in introducing new vaccines, and public engagement could identify concerns that require attention and foster trust to ensure continuous support for immunization [16]. A discrete choice experiment (DCE) can be utilized to determine the rational criteria for prioritization, assess their relative importance (RI), and rank candidates [17]. Although DCEs have been commonly used to investigate public preferences for vaccination, less attention has been given to funding priorities among various vaccines. Luyten et al analyzed the preferences of the population in the United Kingdom [18] and Belgium [19] regarding the inclusion of new vaccines. However, it should be acknowledged that distinct national contexts may influence decision-making criteria valued by the public. Furthermore, we need to assess the priority of candidate vaccines for inclusion in China’s NIP.

Therefore, this study aimed to identify public preferences for including vaccines in the NIP using a DCE and to determine the Chinese population’s desired prioritization of vaccine funding. The findings could aid decision makers in overcoming substantial obstacles when allocating limited resources for incorporating new vaccines into the NIP.

## Methods

The DCE for this study was conducted following current guidelines and recommendations [20-22]. The key steps included defining the research question, identifying attributes and levels, constructing choice tasks, collecting data, and analyzing data.

### Identifying Attributes and Levels

To establish the attributes and levels presented to participants, potential attributes were extracted from previous literature, including those previously used in DCEs for the introduction of new vaccines [18,19], as well as the criteria used in various countries [23] or recommended by the WHO [6] for vaccine introduction. A detailed list of 26 candidate attributes was compiled in Multimedia Appendix 1. Subsequently, a focus group discussion with 20 members of the general public in Nanjing assessed and ranked these candidate attributes, reducing the number to 7 attributes (Multimedia Appendix 2). This was followed by in-depth interviews with 4 DCE participants and 4 vaccine professionals, resulting in the removal of 1 candidate attribute (Multimedia Appendix 3). Table 1 shows the 6 attributes and their corresponding levels. The vaccinated group comprised 4 levels, while the remaining attributes consisted of 3 levels. The establishment of ceiling and baseline levels for these attributes depended on the reported characteristics of prevalent non-NIP vaccines in China, supplemented by an additional level approximating the median value.

**Table 1.** Attributes and levels of vaccines in the discrete choice experiment.

Attributes	Levels
Incidence of vaccine-preventable disease (per 100,000)	10 500 1000
Mortality of vaccine-preventable disease (per 100,000)	0 50 100
Vaccine effectiveness, %	20 50 90
Vaccine cost for all doses (CNY^a^)	100 1000 2000
Vaccinated group	Preschoolers (≤5 years) School-aged children (6‐17 years) Adults (18‐59 years) Older adults (≥60 years)
Vaccine coverage, %	1 30 60

a CNY: Chinese yuan, US $1=7.12 CNY.

### Experimental Design

The identified attributes and levels generated 30 choice sets divided into 3 blocks to reduce the cognitive burden. Each choice set was presented with unlabeled, pairwise, hypothetical vaccine options. The unlabeled design was used to minimize bias that could arise from preexisting perceptions or knowledge about specific vaccines, so that participants would focus on evaluating the impact of the attributes independently. An opt-out option was included to accommodate a vaccine that should not be included in the NIP. A D-efficiency design was utilized to generate the choice sets using Ngene software (version 1.3.0; ChoiceMetrics). The parameters used for the design were primarily derived from the research conducted by Ma et al [15]. The design included a constraint where mortality must be lower than incidence to avoid creating unrealistic choice sets. The mean D-error of design reported by the Ngene software was 0.286.

**Figure 1.**
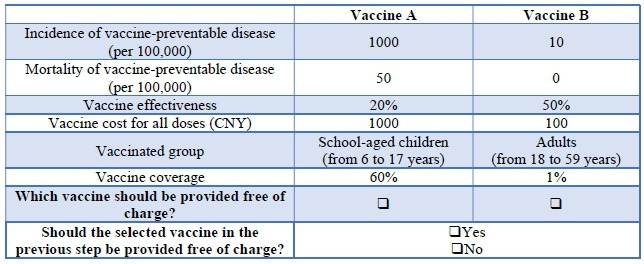
An example of a choice set presented to participants. CNY: Chinese yuan, US $1=7.12 CNY.

A dual-response format [24] was used in the study. Participants initially were required to choose between 2 hypothetical vaccines without an opt-out option (ie, a forced-choice task). Subsequently, they were presented with an opt-out option for the previously selected vaccine from the forced task (ie, an unforced-choice task). Figure 1 displays an example of a choice set. The second choice set was replicated and referred to as the 11th choice set in each block to verify response consistency. The data analysis was focused on the responses to the first 10 choice sets. A randomized order of attributes within the choice sets was used to mitigate potential bias.

Other than the choice sets, the questionnaire also included definitions of attributes and levels, a comprehension test, and sociodemographic inquiries. The sociodemographic information obtained from the inquiries included gender, age, education, income, health status, presence of children, and experience with non-NIP vaccinations. In September 2023, a pilot test was performed involving 30 participants to assess the comprehensibility of the questionnaire. Despite the explanations provided within the questionnaire, the participants in the pilot test faced challenges in understanding NIP and preferred to regard the NIP vaccines as free vaccines. Hence, the relevant descriptions in the questionnaire were adjusted accordingly.

### Data Collection

The sample size was determined using the rule of thumb proposed by Johnson and Orme, (n>500×c/(*t*×a)) [25], which required at least 100 respondents (with a maximum of 4 levels, 10 choice tasks, and 2 alternatives). We selected a target sample size of 1200 to ensure the exclusion of unqualified questionnaires and enhance the reliability of the findings.

The recruitment process for our study was conducted through a detailed and rigorous approach to ensure representativeness. The initial step involved the selection of the sample database. The Wenjuanxing platform was chosen for its prominence as an online survey platform in China and its inclusion of over 2.6 million individuals with verified personal information. More than 2000 studies have used samples from the Wenjuanxing platform in China, as the sample database is extensive and diverse [26]. From this database, we specifically targeted Chinese residents 18 years and older living in Mainland China as eligible participants. To ensure national representation, quota sampling was used. We set target numbers for various categories based on gender, age group, and geographic region, which were aligned with national demographic statistics in the 2020 China Census. Invitations were randomly sent online to these eligible individuals. During the recruitment process, we continuously monitored the incoming responses. If the composition of responses from a particular gender, age group, or region deviated from the quota, we adjusted the invitation sending strategy to focus more on inviting individuals from the underrepresented category. This process continued until we met the predetermined sample size and quota requirements. All participants were recruited indirectly through the platform, and a predetermined fee was charged for recruiting the specified number of eligible participants. Since the platform has such a large and diverse sample database, along with our strict quota sampling method, it effectively facilitated generating an authentic, diverse, and representative sample from all regions of China.

The survey was conducted anonymously using the most prominent online survey platform in China, the Wenjuanxing platform [27]. Participants were informed that the survey aimed to understand their preferences for additional vaccines in the NIP at the start of the survey. They were also provided with information about the vaccines included in the NIP and some commonly used non-NIP vaccines. The survey was conducted between November and December 2023.

In the main analysis, questionnaires were excluded based on three criteria: (1) failure in the comprehension test; (2) a consistent selection of the same answer for each choice (either consistently choosing the left or right option); (3) provision of inconsistent responses in repeated choice sets. In the sensitivity analysis, only the first exclusion criterion was retained, while the others were removed. The sensitivity analysis in our study aimed to evaluate the robustness of our findings using a more lenient set of exclusion criteria. In the main analysis, we applied a strict set of exclusion criteria to ensure the quality of the data. In the sensitivity analysis, we relaxed these criteria. We reincluded responses that consistently selected the same answer for each choice task, as well as inconsistent responses within repeated choice sets, acknowledging that both response types may be plausible.

### Statistical Analysis

Mixed logit (MIXL) models were used to analyze both forced-choice and unforced-choice data. In the case of unforced-choice data, an alternative-specific constant was incorporated into the model to identify the utility of the opt-out option compared to inclusion of a new vaccine. All attributes were treated as categorical variables with dummy coding. Random parameters were estimated using 1500 standard Halton draws, which were determined by incrementally increasing the number of random draws until the model estimates achieved stability (Multimedia Appendices 4 and 5) [28].

The RI for each attribute was calculated by dividing the difference between the coefficients for the most favorable and least favorable levels by the sum of all the attribute differences [29]. The RI of most favorable attribute (vaccine effectiveness) was set at 100. Subgroups were analyzed according to gender, age, region, education, income, health status, presence of children, age of children, and experience with non-NIP vaccines under unforced-choice settings. Significant interaction terms were incorporated into a MIXL model under unforced-choice settings, after all potential interactions between participant characteristics and attribute levels were examined.

The vaccine prioritization for inclusion in the NIP was assessed based on selection probabilities. The selection probability represents the proportion of public support for including the vaccine in the NIP compared to the base case and was calculated using vaccine characteristics and the model estimates. Considering non-NIP vaccines that are currently used in China along with WHO recommendations, a list of candidate vaccines was given, including the HPV vaccine, Hib vaccine, PCV, rotavirus vaccine, varicella vaccine, influenza vaccine, and enterovirus 71 (EV71) vaccine. Recent research findings related to 6 attributes of these candidate vaccines were collected with a focus on the Chinese population and health care system. For the influenza and HPV vaccines, selection probabilities were calculated separately for the different vaccination groups. In contrast, other candidate vaccines were all targeted at preschoolers, thus having only 1 selection probability. The total cost for vaccines was determined by multiplying the price per dose by the number of doses required. Given that vaccine bidding is typically won by manufacturers offering the lowest price, and vaccine inclusion in the NIP can further reduce vaccine prices due to centralized purchasing [30], the current lowest price and price reductions to 50% and 30% of the current lowest price were considered. A MIXL model was constructed to determine the vaccine’s selection probability, with all attributes treated as continuous variables except for the “vaccinated group.” The base case consisted of the least favorable levels of all attributes estimated in the model, including an incidence of 10 per 100,000, mortality of 0, vaccine effectiveness of 20%, cost of 100 Chinese yuan (CNY; US $14.04), vaccination for adults, and coverage of 1%. The 95% CIs were generated using the bootstrap method, while data analysis was performed using Stata (version 16; Stata Corp).

### Ethical Considerations

The study received ethical approval from the Institutional Review Board of Nanjing Medical University (number 2020103). All participants provided written informed consent before joining the survey. The survey was conducted anonymously and individual participants cannot be identified. Participant compensation was provided by the Wenjuanxing platform.

## Results

### Participant Characteristics

A total of 1505 individuals were invited to participate in the survey, with 1258 completing the questionnaire. The average completion time for the questionnaire was 380.77 seconds, and the minimum completion time was 164 seconds. The participants were recruited from 30 provincial-level administrative divisions in China, with 92 failing the comprehension test, 41 consistently choosing the same answer for all choices, and 245 providing inconsistent responses in repeated choice sets. The main analysis was performed with 880 participants, and 1166 were considered for the sensitivity analysis. Table 2 summarizes the characteristics of participants involved in the main analysis, similar to the census-based quota in Multimedia Appendix 6, in terms of gender, age, and region. Of the 880 participants, 50.80% (n=447) were male, 31.93% (n=697) were aged between 45 and 59 years, 79.20% (n=697) had a bachelor’s or college degree, 40.11% (n=353) reported a monthly income ranging from 6000 to 9999 CNY (US $842 to $1403), 56.48% (n=497) were in good health, and a majority of the participants (n=725, 82.39%) had children. A total of 673 participants reported that their children had received non-NIP vaccines, and 659 reported receiving non-NIP vaccines themselves.

**Table 2.** Demographics and characteristics of the final sample in the discrete choice experiment.

Characteristic	Number of participants (n=880), %	
**Gender**		
Male	447 (50.80)	
Female	433 (49.20)	
**Age group (years)**		
18‐29	178 (20.23)	
30‐44	275 (31.25)	
45‐59	281 (31.93)	
≥60	146 (16.59)	
**Region**		
Eastern	413 (46.93)	
Central	228 (25.91)	
Western	239 (27.16)	
**Education**		
Junior high school or below	15 (1.70)	
Senior high school	104 (11.82)	
College	697 (79.20)	
Master or above	64 (7.27)	
**Individual income, CNY/month** ^**a**^		
<3000	77 (8.75)	
3000‐5999	214 (24.32)	
6000‐9999	353 (40.11)	
≥10,000	236 (26.82)	
**Patient-reported health status**		
Very good	177 (20.11)	
Good	497 (56.48)	
Fair	187 (21.25)	
Poor	16 (1.82)	
Very poor	3 (0.34)	
**Age of youngest child (years)**		
0‐5	214 (24.32)	
6‐17	329 (37.39)	
≥18	182 (20.68)	
No children	155 (17.61)	
**Received non-NIP vaccines for your child**		
Yes	673 (76.48)	
No	52 (5.91)	
No children	155 (17.61)	
**Received non-NIP vaccines for yourself**		
Yes	659 (74.89)	
No	221 (25.11)	

a CNY: Chinese yuan, US $1=7.12 CNY.

### Model Estimates

In the unforced-choice settings, model estimates (Table 3) indicated that all 6 attributes significantly influenced the preferences for adopting a new vaccine in the NIP (the *P* value for at least 1 level of each attribute was less than .05). Participants were more likely to choose a vaccine that prevented diseases with higher incidence and mortality, was more effective, and had higher coverage. Under the vaccinated group, preschoolers were given the highest priority, followed by school-age children. The priority levels of vaccinating older adults and younger adults were similar and relatively low (−0.405 vs −0.414). Furthermore, participants preferred that more expensive vaccines be included in the NIP. The coefficient for opt-out was −0.953 (*P*<.001), suggesting that participants demanded more vaccines to be included in the NIP. The estimated standard deviations were mostly significant, indicating heterogeneity in participants’ preferences. Model estimates under the forced-choice settings are listed in Multimedia Appendix 7.

**Table 3.** Estimates of mixed logit models on vaccine preferences under unforced-choice settings.

Attribute and level	Coefficient (95% CI)	*P* value	SD (95% CI)	SD *P* value
**Incidence of vaccine-preventable disease (reference: 10)**				
500	0.053 (−0.094 to 0.199)	.48	0.016 (−0.224 to 0.257)	.89
1000	0.206 (0.050 to 0.362)	.009	0.977 (0.832 to 1.122)	<.001
**Mortality of vaccine-preventable disease (reference: 0)**				
50	0.198 (0.082 to 0.313)	.001	0.674 (0.488 to 0.86)	<.001
100	0.244 (0.083 to 0.405)	.003	1.288 (1.058 to 1.518)	<.001
**Vaccine effectiveness (reference: 20%)**				
50%	0.533 (0.418 to 0.648)	<.001	0.037 (−0.148 to 0.222)	.70
90%	1.095 (0.959 to 1.232)	<.001	1.141 (0.998 to 1.285)	<.001
**Vaccine cost for all doses (reference: 100 CNY)** ^**a**^				
1000 CNY	0.091 (−0.012 to 0.193)	.08	−0.586 (−0.767 to −0.405)	<.001
2000 CNY	0.210 (0.086 to 0.333)	.001	−1.028 (−1.207 to −0.849)	<.001
**Vaccinated group (reference: preschoolers)**				
School-aged children (5‐17 years)	−0.187 (−0.306 to −0.069)	.002	−0.014 (−0.23 to 0.202)	.90
Adults (18‐60 years)	−0.414 (−0.547 to −0.281)	<.001	0.750 (0.536 to 0.963)	<.001
Older adults (≥60 years)	−0.405 (−0.533 to −0.278)	<.001	0.806 (0.619 to 0.993)	<.001
**Vaccine coverage (reference: 1%)**				
30%	0.594 (0.487 to 0.701)	<.001	−0.033 (−0.251 to 0.185)	.77
60%	1.030 (0.899 to 1.161)	<.001	1.093 (0.952 to 1.235)	<.001
**Opt-out**	−0.953 (−1.245 to −0.662)	<.001	2.316 (2.042 to 2.589)	<.001

a CNY: Chinese yuan, US $1=7.12 CNY.

### Attribute RI

In the unforced-choice settings, participants regarded vaccine effectiveness as having the most significant importance (RI=100), followed closely by vaccine coverage (RI=94). However, the importance was comparatively lower in attributes including the vaccinated group (RI=37), mortality of vaccine-preventable disease (RI=22), vaccine cost (RI=19), and incidence of vaccine-preventable disease (RI=19). In the forced-choice setting, both vaccine effectiveness (RI=100) and coverage (RI=87) were also the most important attributes (Table 4).

**Table 4.** Relative importance of vaccine attributes under unforced- and forced-choice settings.

	Unforced setting	Forced setting
Incidence of vaccine-preventable disease	19	23
Mortality of vaccine-preventable disease	22	26
Vaccine effectiveness	100	100
Vaccine cost	19	36
Vaccinated group	37	32
Vaccine coverage	94	87

### Forced-Choice Versus Unforced-Choice Setting

**Figure 2.**
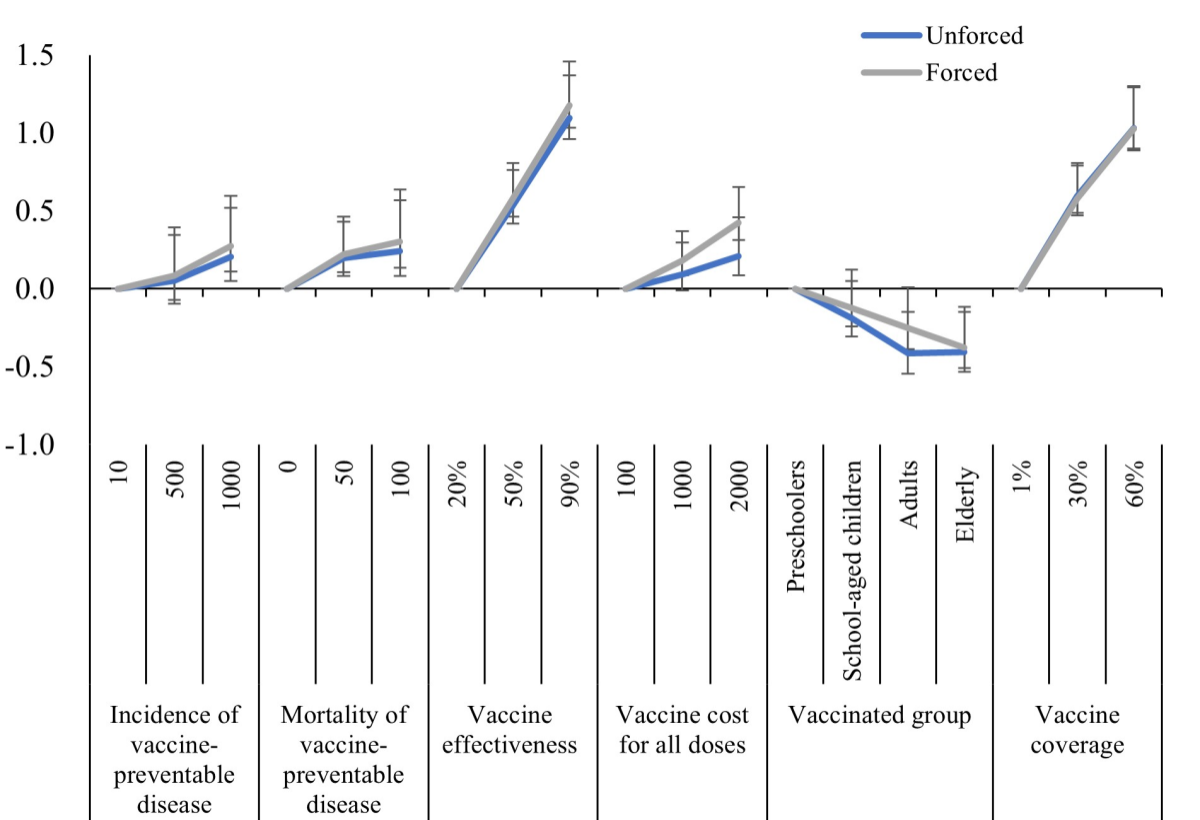
Coefficients of mixed logit models on vaccine preferences under unforced- and forced-choice settings.

The differences in vaccine cost and vaccinated group between unforced- and forced-choice settings were observed by comparing RI and model estimates in Figure 2, respectively. The RI of vaccine cost was lower under the unforced-choice setting (RI=19) when compared with the forced-choice setting (RI=36). The coefficients for both adults and older adults were similar under unforced-choice settings, whereas the coefficients for adults exceeded those of older adults under forced-choice settings.

### Subgroup Analysis

Divergent preference patterns were observed within subgroups for the RI of different vaccine attributes (Multimedia Appendix 8). Subgroup analysis of gender regarding vaccine-preventable diseases showed that males exhibited a greater emphasis on mortality (RI=32) compared to incidence (RI=7). In contrast, females were more concerned about incidence (RI=26) than mortality (RI=13). Participants aged 18‐29 years showed indifference to both incidence and vaccine cost. In the eastern region, vaccine effectiveness was deemed the most important attribute, followed by vaccine coverage, while participants in the central and western regions prioritized vaccine coverage over effectiveness. Participants with a high school education or lower, monthly incomes below 6000 CNY (US $842) or exceeding 10,000 CNY (US $1403), and those in excellent health identified vaccine coverage as the most important attribute. Conversely, participants with a monthly income surpassing 10,000 CNY (US $1403) or in fair or poor health considered vaccine cost the least important among the 6 attributes. Furthermore, participants who had received non-NIP vaccines for themselves or their children ranked vaccine cost as the fourth most important attribute. In contrast, those without such vaccinations considered the cost the least important attribute.

### Interactions

We identified 2 significant interaction terms under the unforced-choice settings (Table 5). One interaction term was observed between the eastern region and vaccine effectiveness of 90%, while the other was found between individuals with good health status (including both very good and good) and vaccine coverage of 60%. Participants from the eastern region were more likely to choose a vaccine with 90% effectiveness than those from the central and western regions (*β*=.374, *P*=.001). Furthermore, individuals with very good and good health status expressed greater concern about 60% vaccine coverage than those with fair, poor, and very poor health status (*β*=.322, *P*=.009).

**Table 5.** Estimates of mixed logit model with main effects and interactions on vaccine preferences.

Attribute and level	Coefficient (95% CI)	*P* value	SD (95% CI)	SD *P* value
**Incidence of vaccine-preventable disease (reference: 10)**				
500	0.038 (−0.108 to 0.184)	.61	0.038 (−0.187 to 0.263)	.74
1000	0.208 (0.052 to 0.364)	.009	1.042 (0.898 to 1.186)	<.001
**Mortality of vaccine-preventable disease (reference: 0)**				
50	0.193 (0.080 to 0.306)	.001	0.630 (0.448 to 0.811)	<.001
100	0.227 (0.071 to 0.384)	.004	1.181 (0.966 to 1.396)	<.001
**Vaccine effectiveness (reference: 20%)**				
50%	0.511 (0.398 to 0.623)	<.001	0.015 (−0.176 to 0.207)	.88
90%	0.899 (0.735 to 1.064)	<.001	1.112 (0.976 to 1.248)	<.001
**Vaccine cost for all doses (reference: 100 CNY)** ^**a**^				
1000 CNY	0.106 (0.005 to 0.207)	.04	0.563 (0.382 to 0.745)	<.001
2000 CNY	0.195 (0.072 to 0.317)	.002	1.010 (0.838 to 1.181)	<.001
**Vaccinated group (reference: preschoolers)**				
School-aged children (5‐17 years)	−0.196 (−0.314 to −0.078)	.001	−0.001 (−0.211 to 0.209)	>.99
Adults (18‐60 years)	−0.425 (−0.557 to −0.294)	<.001	0.708 (0.490 to 0.925)	<.001
Older adults (≥60 years)	−0.406 (−0.532 to −0.280)	<.001	0.783 (0.604 to 0.963)	<.001
**Vaccine coverage (reference: 1%)**				
30%	0.592 (0.487 to 0.698)	<.001	−0.008 (−0.228 to 0.212)	.95
60%	0.774 (0.559 to 0.989)	<.001	0.940 (0.718 to 1.162)	<.001
**Opt-out**	−1.029 (−1.323 to −0.735)	<.001	2.395 (2.131 to 2.659)	<.001
**Interaction term**				
Eastern_effectiveness90%	0.374 (0.162 to 0.586)	.001	−0.002 (−0.693 to 0.690)	>.99
Good health_coverage60%	0.322 (0.080 to 0.563)	.009	−0.631 (−1.007 to −0.255)	.001

a CNY: Chinese yuan, US $1=7.12 CNY.

### Sensitivity Analysis

In the unforced-choice settings, the model estimates (Multimedia Appendix 9) and attribute RI values (Multimedia Appendix 10) remained consistent with those obtained in the main analysis, even when including questionnaires that consistently chose the same response or provided inconsistent responses in repeated choice sets. Additionally, the coefficient for the opt-out option retained its negative value.

### Priority of Candidate Vaccines

The probability of including the vaccine in the NIP (Table 6) was calculated using the evidence for candidate vaccines and the model estimates with all attributes except vaccinated group coded as continuous variables (Multimedia Appendix 11). The varicella vaccine demonstrated the highest selection probability (0.954, 95% CI 0.939‐0.970), suggesting that it should be given the highest priority for inclusion in the NIP. This was followed by the Hib vaccine, EV71 vaccine, influenza vaccine for preschoolers, influenza vaccine for school-age children, PCV, HPV vaccine for school-age children, HPV vaccine for adults, influenza vaccine for older adults, influenza vaccine for adults, and rotavirus vaccine. Furthermore, despite price reductions to 30% and 50%, the top 5 vaccines in terms of selection probability remained the same, in the order of varicella vaccine, Hib vaccine, EV71 vaccine, influenza vaccine for preschoolers, and influenza vaccine for school-age children (Multimedia Appendix 12). Although the rankings of PCV and HPV vaccine for school-age children changed when the price was reduced to 30%, their selection probability remained comparable at 0.754 and 0.772, respectively.

**Table 6.** Selection probability of candidate vaccines for inclusion in the National Immunization Program.

Vaccine	Incidence (per 100,000)^a^	Mortality (per 100,000)	Effectiveness, %	Cost for all doses (CNY)^b^ [31]	Coverage, %	Selection probability (95% CI)
Varicella vaccine	55.05 [32]	0.0005 [32]	90 [33]	302.0	67.1 [5]	0.954 (0.939-0.970)
*Haemophilus influenzae* b vaccine	301 [11]	4 [11]	93 [34]	333.6	25.0 [5]	0.904 (0.876-0.932)
Enterovirus 71 vaccine	134.59 [35]	0.03 [35]	89.7 [36]	366.0	23.9 [5]	0.890 (0.859-0.920)
Influenza vaccine for preschoolers	1050 [37]	2.67 [38]	57 [39]	269.0	28.4 [40]	0.863 (0.824-0.902)
Influenza vaccine for school-age children	1050 [37]	2.67 [38]	47 [39]	708.0	25.1 [40]	0.815 (0.765-0.865)
Pneumococcal conjugate vaccine	679 [11]	10 [11]	60.9 [41]	1892.0	5.1 [5]	0.809 (0.754-0.864)
Human papillomavirus vaccine for school-age children	11.34 [42]	3.36 [42]	80.72 [43]	1017.0	2.24 [44]	0.787 (0.734-0.840)
Human papillomavirus vaccine for adults	11.34 [42]	3.36 [42]	80.72 [43]	1017.0	2.24 [44]	0.694 (0.638-0.750)
Influenza vaccine for the elderly	157 [37]	122.79 [38]	18 [39]	1180.0	26.7 [40]	0.629 (0.529-0.729)
Influenza vaccine for adults	429 [37]	2.67 [38]	36 [39]	2478.0	6.7 [40]	0.554 (0.460-0.647)
Rotavirus vaccine	178.1 [45]	0.14 [46]	85 [47]	561.0	1.8 [5]	0.432 (0.400-0.464)

a Data obtained from studies maintained their original precision.

b CNY: Chinese yuan, US $1=7.12 CNY.

## Discussion

### Principal Findings

This study conducted a DCE to analyze Chinese preferences for incorporating vaccines into the NIP. Our findings revealed that vaccine effectiveness and coverage were the most crucial factors in new vaccine inclusion. The vaccinated group and the mortality of vaccine-preventable diseases also played a significant role, surpassing the importance of vaccine cost and incidence of vaccine-preventable diseases. The most preferred vaccine characteristic to incorporate into the NIP, as inferred from the attributes evaluated, aligns with characteristics of the varicella vaccine, followed by the Hib vaccine, EV71 vaccine, influenza vaccine for preschoolers, and influenza vaccine for school-age children. A distinct pattern of preferences was also observed among different populations through analysis of the study.

This study utilized a dual-response design instead of a direct opt-out option to obtain more preference information for comparing results between the forced- and unforced-choice settings [48]. The observed disparity in participant preferences between both settings could be attributed to the participant’s tendency to select an option they may disagree with under the forced-choice setting. In other words, the unforced-choice setting was more effective in drawing authentic preferences from participants. Therefore, adding an opt-out option in a DCE was necessary, and this was consistent with the previous research finding that forced-choice tasks may bias the analysis results [49,50]. Consequently, we used the data obtained in the unforced-choice setting in the following subgroup analyses, interaction effects analyses, and priority evaluations. In addition, we initially treated all attributes as categorical variables in order to better capture preferences at each level. The results showed that all attributes except the vaccinated group were approximately continuous variables. Consequently, to simplify the calculation of selection probabilities, these attributes were treated as continuous variables.

A significant proportion of the participants (more than 70%) were highly educated, which may be attributed to several factors. First, the complexity of a DCE may necessitate a certain level of literacy and understanding, which could be more prevalent among individuals with higher education. Second, individuals with higher levels of education may exhibit a greater interest in participating in research studies and be more comfortable and familiar with online platforms, thus making them more likely to engage in an online survey. It should be noted that this may have impacted the representativeness of the sample in relation to the general population. However, the sample aligns with the national population in terms of gender, age, and region, thereby ensuring representativeness.

The participants in this study believed that vaccine effectiveness and coverage should be the primary factors when considering a new vaccine in the NIP. Vaccine effectiveness was the most important attribute based on previous studies on vaccination decisions [51,52]. Vaccine coverage ranked as the second most important factor, suggesting that the public’s acceptance and demand for the vaccine was also essential when incorporating a new vaccine in the NIP. Furthermore, the public preferred that more expensive vaccines be included in the NIP over cheaper ones. This inclination likely stemmed from the public’s increasing concern about alleviating their personal financial burden. However, the high cost of vaccines often hinders vaccine integration into the NIP due to the government’s limited financial resources. Therefore, finding a harmonious balance between reducing individual financial burdens and ensuring government affordability is crucial.

Our study revealed that the nature of the disease could influence preferences for vaccine inclusion. It is reasonable to consider that high morbidity and mortality rates may indicate a significant threat to community health, requiring greater control through NIP. Similar to the principles and considerations for adding a vaccine to NIP issued by the WHO [6], the disease targeted by the vaccine was a crucial factor to consider. A previous review also identified the burden of disease as a common criterion for introducing vaccines in different countries [23]. Our findings provided insights into the preferences for inclusion of vaccines for different diseases, just as we previously evaluated the prioritization of vaccines for different diseases. Furthermore, the results of this study can be applied to vaccines for diseases with similar incidence and mortality, as vaccine inclusion preferences differ based on vaccine characteristics such as effectiveness and cost.

This study suggested that varicella vaccine should be prioritized for inclusion in the NIP, probably due to its notably higher coverage than other potential vaccines. A previous study found that 84.1% of Chinese public health workers recommended varicella vaccination, thus making it the most recommended non-NIP vaccine in China [53]. The domestic varicella vaccine became available in 2000, earlier than other non-NIP vaccines [54]. Therefore, the extensive promotional efforts undertaken over a prolonged period have significantly enhanced awareness, familiarity, and acceptance of the varicella vaccine among public health workers and the general public. Furthermore, the high vaccine coverage can be attributed to free varicella vaccines provided by local governments, such as Beijing and Jiangsu [55].

The priority of vaccine inclusion in China’s NIP was assessed by 2 previous studies using expert perspectives. Ma et al [15] regarded the top 5 vaccines as varicella vaccine, meningococcal conjugate AC vaccine, Hib vaccine, influenza vaccine, and EV71 vaccine. Our findings aligned with this study for ranking varicella and Hib vaccines as high priorities. However, Zhang et al [14] prioritized PCV, rotavirus vaccine, Hib vaccine, and varicella vaccine. The differences in vaccine priorities may be due to variations in the studied populations, decision-making criteria, and candidate vaccines.

The effectiveness of PCV against community-acquired pneumonia was used as a proxy for the vaccine’s effectiveness. Community-acquired pneumonia is a serious respiratory infection that can lead to severe complications and even death. In contrast, although otitis media is also common, it usually does not cause serious health effects in patients. Therefore, the focus was on community-acquired pneumonia to evaluate the effectiveness of PCV. Additionally, data on the effectiveness of PCV for otitis media in China were lacking, with only data on community-acquired pneumonia available.

### Limitations

This study has several limitations. First, nonprobability sampling was used through an online survey platform, which may affect the sample representativeness. However, quota sampling was used to approximate nationally representative samples based on demographic characteristics. Furthermore, nonprobability samples were considered acceptable for studying relationships between variables, as indicated in the 2013 American Association for Public Opinion Research report [56]. Future studies should consider using probability-based sampling to replicate the findings. Second, the assessment of priority in this study was heavily based on the vaccine characteristics and disease burden reported in previous studies. Thus, future updates regarding evidence related to vaccines may influence the priority ranking in studies. Third, only 6 attributes were included in this study, which may result in the omission of other significant attributes. Nevertheless, excessive attributes in a DCE study may cause cognitive overload among participants. Last, vaccine inclusion into the NIP is a complex process that requires thorough consideration and should not be based solely on public preferences and demand. Future studies should analyze vaccine prioritization from multiple perspectives.

### Conclusion

This study investigated public preferences regarding vaccine inclusion in China’s NIP. The analysis found that vaccine effectiveness and coverage were the most important factors, followed by the vaccinated group and mortality of vaccine-preventable diseases. Furthermore, different preference patterns were identified among subgroups. The varicella vaccine was highly recommended for inclusion in China’s NIP, followed by the Hib vaccine, EV71 vaccine, influenza vaccine for preschoolers, and influenza vaccine for school-aged children. The inclusion of key vaccines in the NIP is critical to achieving the goals of the Immunization Agenda 2030. This study, therefore, provided valuable insights into this issue. In addition, adding an opt-out option in the DCE was necessary.

## Supplementary material

10.2196/57798 Multimedia Appendix 1 Candidate attributes extracted from the literature.

10.2196/57798 Multimedia Appendix 2 Focus group discussion.

10.2196/57798 Multimedia Appendix 3 Interviews with experts.

10.2196/57798 Multimedia Appendix 4 Mixed logit model for the unforced-choice dataset with varying numbers of draws.

10.2196/57798 Multimedia Appendix 5 Mixed logit model for the forced-choice dataset with varying numbers of draws.

10.2196/57798 Multimedia Appendix 6 Quotas and samples.

10.2196/57798 Multimedia Appendix 7 Estimates of mixed logit models under forced-choice settings.

10.2196/57798 Multimedia Appendix 8 Relative importance of attributes in subgroup analysis.

10.2196/57798 Multimedia Appendix 9 Model estimates in the sensitivity analysis.

10.2196/57798 Multimedia Appendix 10 Relative importance of attributes under main and sensitivity analyses.

10.2196/57798 Multimedia Appendix 11 Model estimates for all attributes as continuous variables except for vaccinated group.

10.2196/57798 Multimedia Appendix 12 Selection probability after reducing vaccine price.

## Abbreviations

CNY: Chinese yuan
DCE: discrete choice experiment
EV71: enterovirus 71
Hib: *Haemophilus influenzae* type b
HPV: human papillomavirus
MIXL: mixed logit
NIP: National Immunization Program
PCV: pneumococcal conjugate vaccine
RI: relative importance
WHO: World Health Organization
