# Supplementary material for: Public Preference and Priorities for Including Vaccines in China’s National Immunization Program: Discrete Choice Experiment
Source: JMIR Public Health Surveill. 2024 Nov 14;10:e57798. doi: 10.2196/57798 (PMC11611798; doi:10.2196/57798)
Supplement: Multimedia Appendix 1 [file publichealth-v10-e57798-s001.docx]

**Appendix 1.** Candidate attributes extracted from the literature.

|  | Candidate attributes | Description in the literature | Levels | References |
| --- | --- | --- | --- | --- |
| 1 | Vaccinated group | Age of vaccinated group/targeted age | Children (3 months−3 years), adults (30–50 years), elderly (65–75 years) | [1,2] |
| 2 | Vaccine effectiveness | Disease episodes prevented in vaccinated group/vaccine efficacy or effectiveness | 1000, 3000, and 5000 cases | [1–3] |
| 3 | Vaccine-induced side-effects | Number of vaccine-induced side-effects/vaccine safety | 100, 300, and 500 cases | [1–3] |
| 4 | Disease episodes prevented via herd protection | Disease episodes prevented via herd protection | 1000, 3000, and 5000 cases | [1] |
| 5 | Age of people receiving herd protection | Age of people receiving herd protection | Newborns (< 3 months), adults (30–50 years), elderly (> 80 years) | [1] |
| 6 | Mortality of vaccine-preventable disease | Expected number of deaths (per year)/burden of disease/mortality | 10, 50, 100 | [2–4] |
| 7 | Expected number of patients with lifelong morbidity | Expected number of patients with lifelong morbidity (per year)/burden of disease/ incidence | 100, 500, 1000 | [2–4] |
| 8 | Expected number of patients with short-term morbidity | Expected number of patients with short-term morbidity (per year)/burden of disease/ incidence | 1000, 5000, 10 000 | [2–4] |
| 9 | Hospitalization and disability rates | Hospitalization and disability rates/burden of disease |  | [2,3] |
| 10 | Socio-economic background of those infected | Socio-economic background of those infected | Below poverty  line, above poverty  line | [4] |
| 11 | Costs of disease | Economic impact of the disease/costs of disease | 10 000 €, 30 000 € | [3,4] |
| 12 | Contribution of the program to disease eradication objectives | Contribution of the program to disease eradication objectives | Yes, no | [4] |
| 13 | Scientific certainty on vaccine effectiveness | Scientific certainty on vaccine effectiveness/quality  of evidence | 100% certainty, 75% certainty | [3,4] |
| 14 | Availability of vaccine | Delivery issues/availability of vaccine supply/accessibility |  | [2,3] |
| 15 | Cost-effectiveness | Economic evaluation/cost-effectiveness |  | [2,3] |
| 16 | Cost | Cost/Incremental costs |  | [2,3] |
| 17 | Vaccine price | Vaccine price |  | [3] |
| 18 | Cost-effectiveness of alternatives | Cost-effectiveness of alternatives |  | [3] |
| 19 | Effectiveness of alternatives | Effectiveness of alternatives |  | [2,3] |
| 20 | Acceptability of vaccine | Acceptability of vaccine |  | [3] |
| 21 | Equity and ethics | Equity and ethics |  | [2,3] |
| 22 | Budget impact | Budget impact and affordability/Financial sustainability |  | [2,3] |
| 23 | Number of doses required | Number of doses required |  | [2] |
| 24 | Formulation | Formulation | Combination vs. monovalent products; Lyophilized vs. liquid products; Heat and freeze sensitivity | [2] |
| 25 | Presentation and packaging | Presentation and packaging |  | [2] |
| 26 | Impact on health system | Impact on health system |  | [2] |

**References**

1. Luyten J, Kessels R, Atkins KE, Jit M, Van Hoek AJ. Quantifying the public’s view on social value judgments in vaccine decision-making: a discrete choice experiment. Social Science & Medicine 2019 May;228:181–193. doi: 10.1016/j.socscimed.2019.03.025

2. World Health Organization. Principles and considerations for adding a vaccine to a national immunization program: from decision to implementation and monitoring. Available from: https://www.who.int/publications-detail-redirect/9789241506892 [accessed Aug 17, 2023]

3. Donadel M, Panero MS, Ametewee L, Shefer AM. National decision-making for the introduction of new vaccines: a systematic review, 2010–2020. Vaccine 2021 Apr 1;39(14):1897–1909. PMID:33750592

4. Luyten J, Beutels P, Vandermeulen C, Kessels R. Social preferences for adopting new vaccines in the national immunization program: a discrete choice experiment. Social Science & Medicine 2022 Jun 1;303:114991. doi: 10.1016/j.socscimed.2022.114991
