# Supplementary material for: Public Preference and Priorities for Including Vaccines in China’s National Immunization Program: Discrete Choice Experiment
Source: JMIR Public Health Surveill. 2024 Nov 14;10:e57798. doi: 10.2196/57798 (PMC11611798; doi:10.2196/57798)
Supplement: Multimedia Appendix 2 [file publichealth-v10-e57798-s002.docx]

**Appendix 2.** Focus group discussion.

The focus group began by evaluating the candidate attributes extracted from the literature and subsequently ranking them. Following the focus group discussion, the participants have reached the following consensus:

1. Combining similar or indistinguishable attributes; (1) combining “short-term morbidity” and “lifelong morbidity” into “incidence”; (2) combining “vaccine price” and “cost” into “vaccine cost” and accurately describing it as the “vaccine cost for all doses”, considering the “number of doses required” for different vaccines.

2. Removing the attributes “cost-effectiveness” and “budget impact”. Both attributes overlapped with cost or effectiveness, and the public faced difficulty understanding them. Therefore, both attributes for “cost-effectiveness” and “budget impact” were removed as “cost” and “effectiveness” were already included in the candidate attributes.

3. Introducing the attribute “vaccine coverage”. Participants indicated that a high vaccine coverage reflected high demand for the particular vaccine, and they preferred vaccines with high coverage to be provided free of charge by the government.

The participants ranked the adjusted attributes, and the top seven rankings were listed in Table S1.

**Table S1.** Attribute ranking in focus group discussion.

| Rank | Attributes |
| --- | --- |
| 1 | Vaccine effectiveness |
| 2 | Vaccine-induced side effects |
| 3 | Vaccine coverage |
| 4 | Vaccinated group |
| 5 | Vaccine cost for all doses |
| 6 | Incidence of vaccine-preventable disease |
| 7 | Mortality of vaccine-preventable disease |
