# Supplementary material for: Public Preference and Priorities for Including Vaccines in China’s National Immunization Program: Discrete Choice Experiment
Source: JMIR Public Health Surveill. 2024 Nov 14;10:e57798. doi: 10.2196/57798 (PMC11611798; doi:10.2196/57798)
Supplement: Multimedia Appendix 3 [file publichealth-v10-e57798-s003.docx]

**Appendix 3.** Interviews with experts.

The experts recommended removing the attribute of “vaccine-induced side effects”. Vaccine side effects are extremely rare, and the exaggerated ranking of this attribute in the focus groups may be attributed to the omission. Vaccines generally undergo comprehensive safety evaluations prior to market approval, and safety is of lesser relevance to the specific prioritization challenges faced by policymakers and the actual trade-offs they need to consider when deciding whether to include a vaccine in the NIP.

Furthermore, the definition of the attributes and levels set were verified through interviews with experts.
