# Supplementary material for: Public Preference and Priorities for Including Vaccines in China’s National Immunization Program: Discrete Choice Experiment
Source: JMIR Public Health Surveill. 2024 Nov 14;10:e57798. doi: 10.2196/57798 (PMC11611798; doi:10.2196/57798)
Supplement: Multimedia Appendix 5 [file publichealth-v10-e57798-s005.docx]

**Appendix 5.** Mixed logit model for the forced-choice dataset with varying numbers of draws.

MIXL, mixed logit model.
