# Supplementary material for: Public Preference and Priorities for Including Vaccines in China’s National Immunization Program: Discrete Choice Experiment
Source: JMIR Public Health Surveill. 2024 Nov 14;10:e57798. doi: 10.2196/57798 (PMC11611798; doi:10.2196/57798)
Supplement: Multimedia Appendix 6 [file publichealth-v10-e57798-s006.docx]

**Appendix 6.** Quotas and samples.

| Characteristic | Participants (N = 880) | Quotas ^a^ | *P*-value |
| --- | --- | --- | --- |
|  | % | % |  |
| **Gender** |  |  | 1.00 |
| Male | 50.80 | 51.24 |  |
| Female | 49.20 | 48.76 |  |
| **Age, year** |  |  | 0.68 |
| 18−29 | 20.23 | 17.60 |  |
| 30−44 | 31.25 | 28.41 |  |
| 45−59 | 31.93 | 30.27 |  |
| ≥ 60 | 16.59 | 23.73 |  |
| **Region** ^b^ |  |  | 1.00 |
| Eastern | 46.93 | 46.97 |  |
| Central | 25.91 | 25.87 |  |
| Western | 27.16 | 27.16 |  |

^a^ The quotas were determined based on the 2020 China census, accessible at: http://www.stats.gov.cn/sj/pcsj/rkpc/7rp/zk/indexch.htm.

^b^ The eastern region includes Beijing, Tianjin, Hebei, Shanghai, Jiangsu, Zhejiang, Fujian, Shandong, Guangdong, Hainan, Liaoning, Jilin, and Heilongjiang; the central region includes Shanxi, Anhui, Jiangxi, Henan, Hubei, and Hunan; and the western region includes Inner Mongolia, Guangxi, Chongqing, Sichuan, Guizhou, Yunnan, Shaanxi, Gansu, Qinghai, Ningxia, and Xinjiang.
