# Supplementary material for: Public Preference and Priorities for Including Vaccines in China’s National Immunization Program: Discrete Choice Experiment
Source: JMIR Public Health Surveill. 2024 Nov 14;10:e57798. doi: 10.2196/57798 (PMC11611798; doi:10.2196/57798)
Supplement: Multimedia Appendix 7 [file publichealth-v10-e57798-s007.docx]

**Appendix 7.** Estimates of mixed logit models under forced-choice settings.

| Attribute and level | Coefficient (95% CI) | *P*-value | SD (95% CI) | SD *P*-value |
| --- | --- | --- | --- | --- |
| **Incidence of vaccine-preventable disease (ref: 10)** |  |  |  |  |
| 500 | 0.083 (-0.072, 0.238) | .29 | 0.001 (-0.218, 0.219) | 1.00 |
| 1000 | 0.273 (0.112, 0.435) | .001 | 1.052 (0.897, 1.207) | < .001 |
| **Mortality of vaccine-preventable disease (ref: 0)** |  |  |  |  |
| 50 | 0.224 (0.105, 0.343) | < .001 | 0.877 (0.709, 1.045) | < .001 |
| 100 | 0.301 (0.133, 0.47) | < .001 | 1.431 (1.195, 1.667) | < .001 |
| **Vaccine effectiveness (ref: 20%)** |  |  |  |  |
| 50% | 0.578 (0.464, 0.692) | < .001 | -0.034 (-0.209, 0.142) | .71 |
| 90% | 1.176 (1.034, 1.317) | < .001 | 1.17 (1.026, 1.313) | < .001 |
| **Vaccine cost for all doses (ref: 100)** |  |  |  |  |
| 1000 | 0.182 (0.088, 0.275) | < .001 | -0.086 (-0.469, 0.297) | .66 |
| 2000 | 0.426 (0.312, 0.54) | < .001 | 0.59 (0.408, 0.771) | < .001 |
| **Vaccinated group (ref: preschoolers)** |  |  |  |  |
| School-aged children (5−17 years) | -0.121 (-0.242, 0) | .051 | 0.034 (-0.226, 0.294) | .80 |
| Adults (18−60 years) | -0.254 (-0.386, -0.122) | < .001 | 0.594 (0.354, 0.834) | < .001 |
| Elderly (≥ 60 years) | -0.377 (-0.507, -0.247) | < .001 | 0.903 (0.714, 1.091) | < .001 |
| **Vaccine coverage (ref: 1%)** |  |  |  |  |
| 30% | 0.578 (0.473, 0.684) | < .001 | 0.025 (-0.222, 0.272) | .85 |
| 60% | 1.026 (0.888, 1.164) | < .001 | 1.184 (1.032, 1.336) | < .001 |
