# Supplementary material for: Public Preference and Priorities for Including Vaccines in China’s National Immunization Program: Discrete Choice Experiment
Source: JMIR Public Health Surveill. 2024 Nov 14;10:e57798. doi: 10.2196/57798 (PMC11611798; doi:10.2196/57798)
Supplement: Multimedia Appendix 8 [file publichealth-v10-e57798-s008.docx]

**Appendix 8.** Relative importance of attributes in subgroup analysis.

(a) Grouped by gender

(b) Grouped by age

(c) Grouped by region

(d) Grouped by education

(e) Grouped by income

(f) Grouped by patient-reported health status

(g) Grouped by presence of children

(h) Grouped by the age of youngest child

(i) Grouped by whether the child received a non-NIP vaccine

(j) Grouped by whether the participant received a non-NIP vaccine
