# Supplementary material for: Public Preference and Priorities for Including Vaccines in China’s National Immunization Program: Discrete Choice Experiment
Source: JMIR Public Health Surveill. 2024 Nov 14;10:e57798. doi: 10.2196/57798 (PMC11611798; doi:10.2196/57798)
Supplement: Multimedia Appendix 9 [file publichealth-v10-e57798-s009.docx]

**Appendix 9.** Model estimates in the sensitivity analysis.

| Attribute and level | Coefficient (95% CI) | *P*-value | SD (95% CI) | SD *P*-value |
| --- | --- | --- | --- | --- |
| **Incidence of vaccine-preventable disease (ref: 10)** |  |  |  |  |
| 500 | 0.029 (-0.085, 0.144) | .62 | 0.009 (-0.187, 0.204) | .93 |
| 1000 | 0.119 (0.000, 0.238) | .051 | 0.771 (0.659, 0.884) | < .001 |
| **Mortality of vaccine-preventable disease (ref: 0)** |  |  |  |  |
| 50 | 0.117 (0.029, 0.204) | .009 | -0.553 (-0.701, -0.404) | < .001 |
| 100 | 0.170 (0.050, 0.29) | .005 | -0.933 (-1.108, -0.758) | < .001 |
| **Vaccine effectiveness (ref: 20%)** |  |  |  |  |
| 50% | 0.445 (0.357, 0.534) | < .001 | -0.027 (-0.168, 0.113) | .70 |
| 90% | 0.898 (0.798, 0.999) | < .001 | 0.857 (0.752, 0.963) | < .001 |
| **Vaccine cost for all doses (ref: 100)** |  |  |  |  |
| 1000 | 0.038 (-0.039, 0.115) | .33 | 0.361 (0.178, 0.544) | < .001 |
| 2000 | 0.174 (0.082, 0.265) | < .001 | -0.757 (-0.899, -0.614) | < .001 |
| **Vaccinated group (ref: preschoolers)** |  |  |  |  |
| School-aged children (5−17 years) | -0.131 (-0.224, -0.037) | .006 | -0.019 (-0.188, 0.150) | .83 |
| Adults (18−60 years) | -0.332 (-0.437, -0.227) | < .001 | 0.715 (0.555, 0.875) | < .001 |
| Elderly (≥ 60 years) | -0.351 (-0.452, -0.250) | < .001 | 0.801 (0.661, 0.941) | < .001 |
| **Vaccine coverage (ref:1%)** |  |  |  |  |
| 30% | 0.512 (0.428, 0.596) | < .001 | 0.028 (-0.107, 0.163) | .69 |
| 60% | 0.819 (0.723, 0.916) | < .001 | 0.879 (0.768, 0.990) | < .001 |
| **Opt-out** | -1.282 (-1.529, -1.036) | < .001 | 2.404 (2.176, 2.631) | < .001 |
