# Supplementary material for: Public Preference and Priorities for Including Vaccines in China’s National Immunization Program: Discrete Choice Experiment
Source: JMIR Public Health Surveill. 2024 Nov 14;10:e57798. doi: 10.2196/57798 (PMC11611798; doi:10.2196/57798)
Supplement: Multimedia Appendix 11 [file publichealth-v10-e57798-s011.docx]

**Appendix 11.** Model estimates for all attributes as continuous variables except for vaccinated group.

| Attribute and level | Coefficient (95% CI) | *P-*value | SD (95% CI) | SD *P-*value |
| --- | --- | --- | --- | --- |
| Incidence of vaccine-preventable disease | 0.0003 (0.0002, 0.0005) | < .001 | 0.0011 (0.0009, 0.0013) | < .001 |
| Mortality of vaccine-preventable disease | 0.0026 (0.0005, 0.0046) | .014 | 0.0224 (0.02, 0.0247) | < .001 |
| Vaccine effectiveness | 0.0191 (0.0168, 0.0213) | < .001 | 0.0207 (0.0182, 0.0232) | < .001 |
| Vaccine cost for all doses | 0.0001 (0.0000, 0.0002) | .03 | 0.0011 (0.001, 0.0012) | < .001 |
| Vaccinated group (ref: preschoolers) |  |  |  |  |
| School-aged children (5−17 years) | -0.1079 (-0.2353, 0.0196) | 1.00 | -0.1154 (-0.3637, 0.133) | .36 |
| Adults (18−60 years) | -0.5252 (-0.6742, -0.3761) | < .001 | -1.0231 (-1.2223, -0.8239) | < .001 |
| Elderly (≥ 60 years) | -0.4690 (-0.6097, -0.3282) | < .001 | 1.0072 (0.8252, 1.1893) | < .001 |
| Vaccine coverage | 0.0219 (0.0189, 0.0248) | < .001 | 0.0333 (0.0299, 0.0366) | < .001 |
| Opt-out | -0.5159 (-0.2148, -0.8169) | .001 | -2.4142 (-2.7224, -2.1059) | < .001 |
