# Supplementary material for: Public Preference and Priorities for Including Vaccines in China’s National Immunization Program: Discrete Choice Experiment
Source: JMIR Public Health Surveill. 2024 Nov 14;10:e57798. doi: 10.2196/57798 (PMC11611798; doi:10.2196/57798)
Supplement: Multimedia Appendix 12 [file publichealth-v10-e57798-s012.docx]

**Appendix 12.** Selection probability after reducing vaccine price.

|  | Reduction to 50% | Reduction to 30% |
| --- | --- | --- |
| Varicella vaccine | 0.954 (0.938, 0.970) | 0.953 (0.937, 0.969) |
| Haemophilus influenza b vaccine | 0.902 (0.875, 0.930) | 0.902 (0.874, 0.930) |
| Enterovirus 71 vaccine | 0.887 (0.857, 0.918) | 0.887 (0.856, 0.917) |
| Influenza vaccine for preschoolers | 0.861 (0.821, 0.900) | 0.860 (0.820, 0.899) |
| Influenza vaccine for school-age children | 0.809 (0.758, 0.859) | 0.806 (0.756, 0.856) |
| Pneumococcal conjugate vaccine | 0.790 (0.740, 0.841) | 0.754 (0.707, 0.801) |
| Human Papillomavirus vaccine for school-age children | 0.776 (0.724, 0.828) | 0.772 (0.720, 0.824) |
| Human Papillomavirus vaccine for adults | 0.679 (0.626, 0.732) | 0.673 (0.621, 0.725) |
| Influenza vaccine for the elderly | 0.609 (0.510, 0.708) | 0.601 (0.502, 0.701) |
| Influenza vaccine for adults | 0.505 (0.442, 0.569) | 0.485 (0.432, 0.538) |
| Rotavirus vaccine | 0.425 (0.395, 0.455) | 0.422 (0.393, 0.451) |
